# Measuring and understanding death anxiety in caregivers of patients with primary brain tumor

**DOI:** 10.1017/S1478951522001110

**Published:** 2023-10

**Authors:** Kelcie Willis, Scott G. Ravyts, Autumn Lanoye, Morgan P. Reid, Farah J. Aslanzadeh, Sarah Ellen Braun, Dace Svikis, Gary Rodin, Ashlee R. Loughan

**Affiliations:** 1 Department of Psychology, Virginia Commonwealth University, Richmond, VA; 2 Massey Cancer Center, Virginia Commonwealth University, Richmond, VA; 3 Department of Health Behavior and Policy, School of Medicine, Virginia Commonwealth University, Richmond, VA; 4 Department of Neuropsychology, Baltimore VA Medical Center, Baltimore, MD; 5 Department of Neurology, Virginia Commonwealth University, Richmond, VA; 6 Department of Supportive Care, Princess Margaret Cancer Centre, University Health Network, University of Toronto, Toronto, ON, Canada

**Keywords:** Caregiver, Death anxiety, Death and dying distress scale, DADDS-CG, Neuro-oncology

## Abstract

**Objective.:**

Caregivers of patients with primary brain tumor (PBT) describe feeling preoccupied with the inevitability of their loved one’s death. However, there are currently no validated instruments to assess death anxiety in caregivers. This study sought to examine (1) the psychometric properties of the Death and Dying Distress Scale (DADDS), adapted for caregivers (DADDS-CG), and (2) the prevalence and correlates of death anxiety in caregivers of patients with PBT.

**Methods.:**

Caregivers (*N* = 67) of patients with PBT completed the DADDS-CG, Patient Health Questionnaire (PHQ-9), Generalized Anxiety Disorder (GAD-7), Fear of Cancer Recurrence (FCR-7), and God Locus of Health Control (GLHC). Caregivers’ sociodemographic information and patients’ medical characteristics were also collected. Preliminary examination of the psychometric properties of the DADDS-CG was conducted using exploratory factor analysis, Cronbach’s alpha, and correlations. The prevalence and risk factors of death anxiety were assessed using frequencies, pair-wise comparisons, and correlations.

**Results.:**

Factor analysis of the DADDS-CG revealed a two-factor structure consistent with the original DADDS. The DADDS-CG demonstrated excellent internal consistency, convergent validity with the PHQ-9, GAD-7, and FCR-7, and discriminant validity with the GLHC. Over two-thirds of caregivers reported moderate-to-severe symptoms of death anxiety. Death anxiety was highest in women and caregivers of patients with high-grade PBT.

**Significance of results.:**

The DADDS-CG demonstrates sound psychometric properties in caregivers of patients with PBT, who report high levels of death anxiety. Further research is needed to support the measure’s value in clinical care and research — both in this population and other caregivers — in order to address this unmet, psychosocial need.

## Introduction

A diagnosis of a primary brain tumor (PBT) is often associated with considerable distress. Negative sequelae of the tumor itself and associated treatments are numerous, including: cognitive decline (Allen and Loughan, 2018); personality changes (Janda et al., 2008); seizures and other neurological symptoms (Klein et al., 2016), fatigue and sleep disturbance (Willis et al., 2021), motor impairment (Amidei and Kushner, 2015); and psychological distress (Mainio et al., 2011). These sequelae not only impact patients’ overall quality of life (Cahill et al., 2012) but also translate into significant caregiver burden.

Given the high level of functional impairment associated with PBT, informal caregivers — who are often spouses, parents, adult children, or other closed loved ones (Sacher et al., 2018) — are frequently required to take on new roles with increased responsibility (McConigley et al., 2010). In addition to managing patients’ activities of daily living (Khalili, 2007), caregivers report heightened clinical demands, such as coordinating the patient’s medical care, monitoring changes in symptoms, communicating with healthcare providers, as well as making significant treatment and end-of-life decisions (Schubart et al., 2008). Yet many caregivers report feeling unprepared and untrained to perform these myriad tasks (Schubart et al., 2008; Goebel et al., 2010; Russell et al., 2016). In fact, consistent research demonstrates that caregivers frequently report higher rates of psychological distress than patients themselves (Petruzzi et al., 2013; Baumstarck et al., 2016; Braun et al., 2021). However, because medical care is typically focused on the patient, the needs of caregivers often not considered, assessed, or addressed (Schubart et al., 2008).

One unmet need reported by caregivers of patients with PBT is support for their existential concerns, including death anxiety. Caregivers describe feeling fearful, preoccupied, or depressed by the anticipatory death and dying of their loved one; this is heightened by the lack of curative treatment options, the likelihood of tumor progression, and overall low survival rates in individuals with brain cancer (Cavers et al., 2012; Applebaum and Breitbart, 2013; Deshields and Applebaum, 2015; Applebaum et al., 2016). In a qualitative study, caregivers of patients with PBT described their experience of death anxiety as an “all-consuming preoccupation” (Wideheim et al., 2002), triggered by even minor neurological symptoms in the patient (Adelbratt and Strang, 2000). Caregivers also revealed that despite their desire for conversations about the patient’s mortality and future, such discussions rarely took place with either the patient or the medical team (Kanter et al., 2013). This mirrors findings from a recent international survey of neuro-oncology providers, which found 29–46% of physicians felt uncomfortable dealing with end-of-life issues (Walbert et al., 2015). Given the known interrelationship between caregiver and patient mental health (Braun et al., 2021), increased understanding of death anxiety in caregivers has potential benefits for both caregivers and patients. Therefore, an investigation of caregiver death anxiety is both clinically and empirically warranted.

To date, there are very few quantitative investigations examining death anxiety in caregivers of patients diagnosed with PBT (Applebaum et al., 2016). Most studies in neuro-oncology have used a single item from a larger quality of life scale (e.g., Partner and Carer Supportive Needs Scale) to measure death anxiety (Long et al., 2016). Only one study to date utilized a specific measure of death anxiety (i.e., Death Anxiety Scale) in caregivers of patients with PBT (Braun et al., 2021). However, in this study, death anxiety was measured from the perspective of the caregiver’s fear of their *own* death (as opposed to caregivers’ death anxiety related to the *patient*’*s* impending death) (Braun et al., 2021). There is also no validated measure of caregivers’ death anxiety in other medical populations. The lack of validated instruments for caregivers inevitably limits our understanding of the prevalence of death anxiety as well as relevant risk and protective factors — two essential components in addressing this unmet, psychosocial need.

The overarching purpose of the current study was to validate a measure of death anxiety for use with caregivers of patient with PBT in order to better understand the prevalence and correlates of death anxiety in this population. First, we examined the psychometric properties of a slightly modified measure of death anxiety originally created for *patients* diagnosed with cancer — the Death and Dying Distress Scale (DADDS) (Krause et al., 2015) — among *caregivers* of patients diagnosed with PBT. The DADDS is a promising measure to adapt for caregivers as it was created in the context of advanced cancer, unlike the DAS (Lo et al., 2011). Moreover, a previous investigation of *patients* in neuro-oncology found the DADDS to be psychometrically superior to the DAS (Loughan et al., 2021). We changed the wording to reflect the caregiver’s anxiety about their loved one’s death and conducted an exploratory factor analysis to determine the psychometric properties of this modified scale. We hypothesized that the modified form of the original DADDS (subsequently called DADDS-CG) would demonstrate a similar factor structure to the original patient version of the DADDS and maintain its strong reliability and validity. Second, we analyzed the prevalence and correlates of death anxiety in caregivers of patients with PBT using the DADDS-CG. Given the high levels of emotional distress documented previously (Petruzzi et al., 2013; Baumstarck et al., 2016; Braun et al., 2021), we hypothesized a similar, high rate of death anxiety in caregivers of patients diagnosed with PBT. However, because the current study represents the first attempt to measure death anxiety in this way, no hypotheses were made regarding sociodemographic, medical, or treatment correlates.

## Methods

### Participants and procedure

Using a cross-sectional design, caregivers of patients with PBT completed surveys that assessed death anxiety, depression, anxiety, fear of cancer recurrence, and God locus of health control. Data collection occurred in two waves: First, caregivers of patients with PBT who received care at a National Cancer Institute Designated Cancer Center completed questionnaires in-person during their loved one’s routine neuro-oncology visits (July 2019–August 2019). Data collection was paused due to insufficient staffing from September 2019 to June 2020. The second wave was converted to a virtual format following COVID-19 safety regulations (July 2020–February 2021), and caregivers of patients diagnosed with PBT were recruited from neuro-oncology social media support groups. Ethical approval was granted from the institutional review board (HM20013477), and informed consent was obtained from all study participants. Inclusion criteria were: (1) age 18 or older, (2) caregiver of patient with a PBT diagnosis, and (3) literate in English. Twenty-four in-person caregivers consented to participate. Forty-nine virtual caregivers consented to participate. In total, six participants (four in-person and two virtual) provided incomplete data. Thus, a total of 67 caregivers were included in the final sample.

### Measures

#### Caregiver sociodemographic variables

Caregivers were asked to report basic sociodemographic information: age, gender identity, race, and their relationship to the patient.

#### Patient medical and treatment characteristics

For in-person participants, patient medical (e.g., tumor type, tumor grade, tumor hemisphere, time since diagnosis), and treatment characteristics (e.g., history of surgical resection, cranial irradiation, chemotherapy) were extracted from medical records. For virtual participants, these same variables were self-reported by the caregiver.

#### Death and Dying Distress Scale-Caregivers (DADDS-CG)

The DADDS-CG is a 15-item questionnaire of death anxiety adapted from the DADDS (Lo et al., 2011) that asks respondents to indicate their degree of distress regarding each item on a scale from 0 (no distress) to 5 (extreme distress) (Lo et al., 2011). Items are summed to produce a total score, where scores of 0–24 indicate low death anxiety, 25–46 indicate moderate death anxiety, and 47–75 indicate severe death anxiety (Krause et al., 2015). The instructions and items were modified to ask caregivers to reflect on *their own* feelings regarding *their loved one*’*s* death. For example, rather than “How distressed do you feel that your death and dying may happen with a lot of pain or suffering?” the item read “How distressed do you feel that your loved ones’ death and dying may happen with a lot of pain or suffering?” See Table 2 for the items on the DADDS that were modified for the DADDS-CG.

#### Patient Health Questionnaire-9 (PHQ-9)

The PHQ-9 is a brief, validated self-report measure designed for use in both clinical and research settings to assess severity of depressive symptoms (Kroenke et al., 2001). Possible total scores range from 0 to 36, with higher scores reflecting greater symptom severity. A score of 10 or higher has been established as a cut-point to indicate clinically significant symptoms of depression. Furthermore, scores of 5, 10, 15, and 20 indicate mild, moderate, moderately severe, and severe symptoms of depression, respectively. Cronbach’s alpha (*α* = 0.91) was high.

#### Generalized Anxiety Disorder-7 (GAD-7)

The GAD-7 is a brief, validated self-report measure designed to assess severity of anxiety symptoms in both clinical and research settings (Spitzer et al., 2006). Items are summed to yield a total score ranging from 0 to 21, with higher scores indicative of greater symptom severity. Scores of 5, 10, and 15 correspond to mild, moderate, and severe levels of anxiety, respectively. Cronbach’s alpha (*α* = 0.94) was high.

#### Fear of Cancer Recurrence-7 (FCR-7)

Caregivers’ fear of patients’ cancer recurrence was assessed using the FCR-7, a seven-item self-report measure (Humphris et al., 2018). For six items, respondents are asked to indicate their frequency of agreement on a five-point scale ranging from 1 (not at all) to 5 (all the time). The seventh item is rated on an 11-point scale from 0 (not at all) to 10 (all the time). Scores are summed to yield a total score ranging from 6 to 40, with higher scores signifying greater fear of cancer recurrence. The original validation paper suggests a comparison score of ≥17 is higher than 60% of patients with mixed cancers, and ≥27 is higher than 90% of patients with mixed cancers (Humphris et al., 2018). As with the DADDS, the instructions and items were modified to reflect the caregivers’ perspective. For example, the original item “I am afraid that my cancer may recur” was changed to “I am afraid that my loved one’s cancer may recur.” Cronbach’s alpha (*α* = 0.88) was high.

#### God Locus of Health Control (GLHC)

The GLHC assess the extent to which the respondent believes that their health is determined or controlled by God (in contrast to internal factors or chance, for example) (Wallston et al., 1999). The GLHC consists of 6 items, each of which ask respondents to indicate the degree of agreement on a six-point scale ranging from 1 (strongly disagree) to 6 (strongly agree). Item scores are summed to yield a total score, ranging from 6 to 36; higher scores indicate greater belief that God is in control of the respondent’s health. However, as with the DADDS and FCR, the wording of items was changed such that responses reflect caregiver belief that God is in control of their loved one’s disease. For example, the original item “God is in control of my condition” was changed to “God is in control of my loved one’s condition.” Cronbach’s alpha (*α* = 0.97) was high.

### Data analysis

In order to determine the factor structure of the DADDS among caregivers, an exploratory factor analysis (EFA) with no *a priori* factor structure was performed using principal axis factoring and a Promax rotation. In accordance with previously established guidelines (Worthington and Whittaker, 2006; DeVellis, 2016), an item was considered to load onto a specific factor if it achieved simple structure, defined as the highest loading eigenvalue exceeding an absolute value of 0.30, with all cross-loadings being at least 0.15 less than the item’s highest factor loading. Internal consistency was assessed via Cronbach’s alpha. Convergent validity was assessed by examining the correlation between participants’ DADDS scores and their scores on the PHQ-9, GAD-7, and FCR-7; these measures were used to assess convergent validity in a previous investigation of patient death anxiety in PBT (Loughan et al., 2021). Divergent validity was assessed by examining the correlation between participants’ scores on the GLHC and the DADDS. The GLHC was chosen given previous research with this latent variable (Krause, 2005) and the potential protective nature of both religiosity (Soleimani et al., 2020) and locus of control (Thorson and Powell, 1988; Brown et al., 2015). Frequency was used to determine the prevalence of death anxiety using the DADDS-CG. Pair-wise comparisons and bivariate correlations were used to determine whether death anxiety among PBT caregivers varied as a function of sociodemographic, medical, or treatment variables.

## Results

In-person and online data collection samples were comparable across age, gender, and ethnicity. A diagnosis of high-grade tumor was significantly more common among virtual participants than in-person participants: 80% vs. 25%; *χ*^2^ (1, *N* = 55) = 7.081, *p* < 0.05. The two samples were comparable across all other medical and treatment variables. The virtual data collection group reported higher psychological distress, including greater generalized anxiety, depression, death anxiety, and fear of cancer recurrence ( *p*s < 0.05) than in-person participants. As such, data collection method was used as a covariate to verify any significant differences found.

### Descriptive statistics

Participants consisted of 67 caregivers of patients with PBT, who were predominantly middle-aged (*M* = 50.61, *SD* = 13.56), women (73.13%), and White (89.55%). Over half of the sample consisted of spouses/partners (59.70%). Nearly one third of the sample endorsed moderate-to-severe (33.30%) levels of anxiety according to the GAD-7. Nearly one fifth of participants (19.40%) endorsed moderate-to-severe levels of depression on the PHQ-9. When compared to a mixed-cancer sample, most caregivers (80%) endorsed fear of their loved one’s tumor recurrence over the >60th percentile and nearly half (48%) over the >90th percentile on the FCR-7. Complete descriptive and clinical statistics are presented in Table 1.

### Psychometric properties of the DADDS-CG among PBT caregivers

KMO (0.87) and Bartlett’s tests (*X*^2^ = 831.69, *df* = 105, *p* < 0.001) for the DADDS-CG suggested that the data were suitable for factor analysis. The EFA produced a scree plot with a pronounced elbow at the second eigenvalue indicating a two-factor structure. Combined these two factors explained 68.39% of the data with the first factor accounting for 56.62% of the data and the second factor accounting for an additional 11.77% of the data. The correlation among both factors was 0.61. Item loadings for these two factors are presented in Table 2.

Cronbach’s alpha was 0.94 for Factor 1, 0.92 for Factor 2, and 0.94 for the full scale indicating excellent internal consistency. For convergent validity, death anxiety among caregivers was positively correlated with depression (*r* = 0.58), anxiety (*r* = 0.57), and fear of cancer recurrence (*r* = 0.68; *p*s < 0.001), as measured by the PHQ-9, GAD-7, and FCR-7, respectively. By contrast, death anxiety on the DADDS-CG was negatively associated with the GLHC (*r* = −0.29, *p* < 0.001). All correlations remained significant following data collection covariation ( *p* < 0.05).

### Prevalence and differences in death anxiety in PBT caregivers

Participants’ mean death anxiety fell within the moderate range (*M* = 34.81, *SD* = 18.96) with 31.34%, 40.30%, and 28.36% of caregivers reporting low, moderate, and severe levels of death anxiety, respectively. The mean score per item for Factor 1 was 2.19 (*SD* = 1.34) while the mean score per item for Factor 2 was 2.59 (*SD* = 1.49). Women were significantly more likely than men to endorse higher levels of death anxiety; *t*(65) = 2.21, *p* < 0.05, Cohen’s *d* = 0.62. Additionally, caregivers of patients with high-grade tumors were also more likely to endorse greater death anxiety; *t*(53) = −2.08, *p* < 0.05, Cohen’s *d* = 0.57. Tumor grade differences remained significant following data collection covariation ( *p* < 0.05). Differences in death anxiety among other sociodemographic, medical, or treatment variables were non-significant. The complete results are presented in Table 3.

## Discussion

Although the focus of treatment is typically on the patient, caregivers also experience major life alterations and significant emotional distress, including death anxiety (Goebel et al., 2010; Applebaum et al., 2016; Russell et al., 2016), that can adversely affect their quality of life (Braun et al., 2021). Validated tools are needed to assess distress of various kinds and to inform the implementation of psychosocial interventions in caregivers. The current study is a preliminary evaluation of the psychometric properties of an existing measure of death anxiety — the Death and Dying Distress Scale — adapted for use with caregivers of patients with PBT (DADDS-CG). We then examined the prevalence and correlates of death anxiety in this sample.

### Validation of the DADDS-CG

In line with our prediction, findings of the present study suggest that the DADDS-CG is a psychometrically sound tool for assessing caregivers’ anxiety about their loved one’s death and dying following a simple language modification to the original DADDS. The DADDS-CG demonstrated strong internal consistency, similar to the original DADDS (Lo et al., 2011). Moreover, the factor structure of the DADDS-CG within caregivers mirrors the factor structure of the DADDS among patients with advanced cancer, in which ten items measure distress about *finitude*, or the shortness of the patient’s remaining time, and five items measure distress about the actual process of the patient’s *dying* (Shapiro et al., 2021). Examination of correlations between the newly developed DADDS-CG and other measures of psychological distress suggests death anxiety is a related but separate construct in caregivers. As expected, the DADDS-CG was moderately correlated with measures of depression and anxiety suggesting good construct validity. Additionally, the DADDS-CG was strongly associated with another measure of cancer-related existential distress — fear of cancer recurrence — providing further evidence of convergent validity. In terms of divergent validity, the DADDS-CG was negatively associated with a measure of religiosity (i.e., the God Locus of Health Control), which is an identified protective factor of death anxiety in previous studies of oncology patients (Soleimani et al., 2020). Given these promising characteristics, the DADDS-CG represents the first measure of death anxiety specific to the *patient*’*s* death validated for use in caregivers of any cancer population.

### Prevalence of death anxiety

Given that the DADDS-CG demonstrated similar psychometric properties as the DADDS, the previously established cutoffs were applied to caregivers of patients with PBT. More than two-thirds (69%) of the caregivers in our sample endorsed moderate-to-severe death anxiety. Moreover, when using the moderate-to-severe clinical cutoff, caregivers endorsed death anxiety at a greater frequency than both depression (19%) and anxiety (33%). This finding parallels a study of patients with PBT, where cluster analysis revealed that most patients endorsed high death anxiety but low emotional distress (i.e., symptoms of depression and anxiety) (Loughan et al., 2020). Yet, death anxiety in caregivers was even more prevalent (69%) than what was identified in a previous sample of patients with PBT, which found that 48% of patients endorsed moderate-to-severe death anxiety on the DADDS (Loughan et al., 2021). This finding echoes caregivers’ description of their loved one’s death as an “all-consuming preoccupation” (Applebaum et al., 2016) and follows a similar pattern of results of other measures of psychological distress, such that caregivers consistently report greater emotional distress than patients with PBT (Petruzzi et al., 2013; Baumstarck et al., 2018; Braun et al., 2021). Overall, this exorbitantly high rate of death anxiety in caregivers of patients with PBT emphasizes the need for greater attention to caregivers’ mental health.

### Correlates of death anxiety

Lastly, we analyzed the sociodemographic, medical, and treatment correlates of death anxiety in caregivers of patients with PBT. We found that there was a high prevalence of this distress across most demographic groups, suggesting that death anxiety was fairly diffuse in the current sample. Consistent with a previous analysis of caregivers in oncology, women were more likely to endorse death anxiety than men (Soleimani et al., 2017); however, it is important to note that this previous investigation assessed caregivers’ death anxiety about their *own* death. Women with advanced cancer have also exhibited greater death anxiety, but this has not been replicated in patients diagnosed with PBT specifically (Loughan et al., 2021; Shapiro et al., 2021). Though the caregiving experience can vary greatly across brain tumor types (Schubart et al., 2008). we found very few medical correlates of death anxiety in the current study. Only caregivers of patients with high-grade tumors endorsed greater death anxiety compared to caregivers of patients with low-grade tumors. Perhaps this is because they are caring for patients with poorer prognoses, whose possible lower functional status serves as a reminder of their mortality (Adelbratt and Strang, 2000). Interestingly, this finding is in contrast to reports from patients with PBT, in which there was no association between death anxiety and tumor grade (Loughan et al., 2021). Caregiver death anxiety was not associated with age, race, relationship to the patient, time since diagnosis, tumor type, tumor hemisphere, or treatment type. However, these findings warrant future corroboration, especially given certain methodological limitations, including caregivers’ self-report of patients’ medical characteristics during online data collection. Nevertheless, the identified high prevalence of death anxiety in the current sample suggests that death anxiety may, in fact, be a ubiquitous experience for many caregivers of patients with PBT, regardless of the patient’s medical or the caregiver’s sociodemographic characteristics.

### Study limitations

The current study provides valuable information regarding the validity of the DADDS-CG and the prevalence and correlates of death anxiety in a unique population; nevertheless, there are a few considerations that would strengthen our findings. One limitation is the sample size. While PBT represents a rare disease for data collection, future confirmatory factor analyses (CFA) of the DADDS-CG will require a much larger sample. Expanding future data collection across institutions might also prove beneficial. While the hybrid recruitment method (i.e., in-person and virtual) due to COVID-19 was not optimal, one benefit was that it allowed for a broader range of participant involvement, potentially capturing more caregivers seeking support (e.g., those from support groups, listservs, social media platforms) than the typical convenience sample of a local neuro-oncology clinic. Another limitation of the current study is the homogeneity of the sample. The vast majority of participants identified as White, middle-aged women. While this is consistent with prior samples of caregivers of patients with PBT (Sacher et al., 2018), future research would benefit from a more diverse array of caregivers — given the known racial and ethnic disparities in cancer care — and extending findings to caregivers of other cancers or terminal diseases (Wilcox and Boire, 2020). The initial development of a death anxiety measure for caregivers of patients with PBT addresses a critical scientific and clinical gap; however there is an opportunity to generalize findings to other caregiving populations in need during subsequent CFA. Next, while the prevalence of death anxiety provides vital, preliminary information, more research is needed regarding the course of death anxiety in caregivers over time, from diagnosis to bereavement. Longitudinal analyses may also reveal distinct trajectories across different sociodemographic, medical, or treatment variables. Lastly, a greater understanding of the dyadic relationship between patient and caregiver death anxiety and related outcomes is needed to improve patient quality of life and subsequent care.

### Clinical implications

The results of the current study have important implications for medical providers, who have previously reported feeling uncomfortable discussing end-of-life issues with families affected by PBT (Walbert et al., 2015). These providers might utilize both the DADDS and DADDS-CG to initiate conversation with families and refer affected individuals to appropriate providers (e.g., psychologists, social workers, chaplains, or other palliative care clinicians), paying closer attention to women caregivers or caregivers of those with high-grade PBT. The high frequency of death anxiety and its association with other quality of life outcomes suggests caregivers are in need of psychosocial intervention to alleviate symptoms. Such interventions may also have benefit for patients, in view of the known interrelationship of patient and caregiver mental health (Braun et al., 2021). Indeed, a closer examination of the items of the DADDS and DADDS-CG reveals that while caregivers are most concerned about their loved one’s death happening “with a lot of pain or suffering,” patients are more concerned about “being a burden” and “the impact” of their death on their “loved one” (Loughan et al., 2021; Shapiro et al., 2021). Therefore, treatments that are able to engage both patients and caregivers in conversations about the disease and dying process, such as Managing Cancer and Living Meaningfully (CALM; Hales and Rodin, 2021) are particularly well positioned to meet interrelated existential needs within the dyad. If dyadic work is not possible, Meaning-Centered Psychotherapy for Cancer Caregivers (MCP-C), which explicitly targets existential concerns, may be another avenue for alleviating death anxiety in this unique population (Applebaum et al., 2022).

## Conclusion

The findings of the present study suggest that DADDS-CG is a valid measure of death anxiety in caregivers of patients with PBT and may have utility in both research and clinical care. High levels of death anxiety were found in our sample, with more than two-thirds of caregivers endorsing moderate-to-severe death anxiety. Additionally, death anxiety was highest in women and those who care for patients with high-grade tumors. Further research with a larger sample size is needed confirm these findings and to perform confirmatory factor analysis. Subsequent research should also examine: (1) the validity of the measure in caregivers of patients with other cancers and other medical conditions, (2) the longitudinal trajectory of this symptom, (3) its relationship to death anxiety in the patient, and (4) its responsiveness to therapeutic interventions. Though the majority of care necessarily focuses on the patient with PBT, greater attention to caregiver death anxiety and intervention is clearly warranted.

## Figures and Tables

**Table 1.** Characteristics of caregivers of patients diagnosed with PBT (*N* = 67)

Variable	Frequency	Mean (SD)/Percent
Age	67	50.61 (13.56)
Gender		
Male	18	26.9%
Female	49	73.1%
Race		
White	60	89.6%
Black	6	9%
Other	1	1.5%
Relationship to Patient		
Partner/Spouse	40	59.7%
Family Member	26	38.8%
Friend	1	1.5%
Tumor Type		
Meningioma	7	12.1%
Astrocytoma	8	13.8%
Oligodendroglioma	9	15.5%
Glioblastoma Multiforme	24	41.4%
Other/Unknown	10	17.2%
Tumor Grade		
High (Grades 1 and II)	37	67.3%
Low (Grades III and IV)	18	32.7%
Tumor Hemisphere		
Left	26	44.8%
Right	24	41.4%
Both	8	13.8%
Time Since Diagnosis		
> 1 year	14	27.5%
1–3 years	14	27.5%
3–5 years	3	5.9%
5–10 years	12	23.5%
10+ years	8	15.7%
Resection		
Yes	45	77.6%
No	13	22.4%
Chemotherapy		
Yes	48	82.8%
No	10	17.2%
Radiation		
Yes	53	91.4%
No	5	8.6%
DADDS-CG	67	34.81 (18.96)
PHQ-9	66	7.21 (6.33)
GAD-7	66	7.76 (6.33)
FCR-7	64	25.31 (8.17)
GLHC	67	17.70 (10.23)

SD, Standard Deviation; DADDS-CG, Death and Dying Distress Scale for Caregivers; PHQ-9, Patient Health Questionnaire-9; GAD-7, Generalized Anxiety Disorder-7; FCR-7, Fear of Cancer Recurrence-7; GLHC, God Locus of Health Control.

**Table 2.** Psychometric properties of the DADDS in caregivers (DADDS-CG)

	Mean	SD	Item-Total Correlation	Factor 1 “Finitude”	Factor 2 “Dying”
*Instructions: Please tell us how distressed you felt over the past two weeks about each item listed below reqardinq **YOUR LOVED ONE***.					
1. Not having done all the things that they wanted	2.34	1.50	0.74	**0.77**	0.07
2. Not having said all that they wanted to say to the people they care about	1.58	1.54	0.73	**0.83**	−0.01
3. Not having achieved their life goals and ambitions	1.94	1.59	0.68	**0.75**	0.03
4. Not knowing what happens near the end of life	2.09	1.73	0.77	**0.80**	0.07
5. Not having a future	2.28	1.77	0.79	**0.89**	−0.03
6. The missed opportunities in their life	2.16	1.67	0.73	**0.87**	−0.06
7. Running out of time	2.36	1.71	0.79	**0.80**	0.07
8. Being a burden to others	1.87	1.78	0.52	**0.82**	−0.24
9. The impact of their death on their loved ones	2.69	1.62	0.77	**0.64**	0.24
*Instructions: Over the post two weeks, how distressed did you feel that **YOUR LOVED ONE S** death ond dying may:*					
10. Their death and dying	2.54	1.74	0.74	**0.87**	−0.06
11. Happen suddenly or unexpectedly	2.40	1.73	0.69	−0.02	**0.91**
12. Be prolonged or drawn out	2.79	1.68	0.64	−0.08	**0.90**
13. Happen when 1 am alone	2.36	1.73	0.61	−0.003	**0.79**
14. Happen with a lot of pain or suffering	3.03	1.74	0.70	−0.06	**0.94**
15. Happen very soon	2.37	1.79	0.71	0.13	**0.75**

Bold numbers represent eigen values loading onto each factor.

**Table 3.** Average death anxiety by sociodemographic, medical, and treatment characteristics

Variable	Mean DADDS-CG (*SD*)	*p*-value
Gender		
Male	26.61 (17.12)	**0.03**
Female	37.82 (18.86)	
Race		
White	33.58 (18.38)	0.22
Black	43.50 (23.67)	
Relationship		
Spouse/Partner	33.25 (19.74)	0.62
Family Member	37.50 (18.08)	
Friend	27 (N/A)	
Patient Diagnosis		
Glioblastoma Multiforme	39.58 (17.55)	0.35
Astrocytoma	36.63 (22.39)	
Oligodendroglioma	30.11 (16.17)	
Meningioma	31.14 (14.90)	
Other	26.50 (16.40)	
Tumor Grade		
High	37.97 (15.96)	**0.04**
Low	27.61 (19.98)	
Tumor Hemisphere		
Left	38.35 (16.05)	0.49
Right	32.54 (18.53)	
Bilateral	32.88 (22.56)	
Resection		
Yes	35.07 (17.79)	0.92
No	35.62 (19.25)	
Chemotherapy		
Yes	36.83 (17.79)	0.13
No	27.30 (17.45)	
Radiation		
Yes	36.19 (17.56)	0.17
No	24.60 (20.70)	
Variable	*r*	*p*-value
Age	−0.07	0.57
Time Since Diagnosis	−0.11	0.43
PHQ-9	0.58	**0.001**
GAD-7	0.57	**0.001**
FCR-7	0.68	**0.001**
GLHC	−0.29	**0.02**

SD, Standard Deviation; DADDS-CG, Death and Dying Distress Scale for Caregivers; PHQ-9, Patient Health Questionnaire-9; GAD-7, Generalized Anxiety Disorder-7; FCR-7, Fear of Cancer Recurrence-7; GLHC, God Locus of Health Control.

The bolded values are statistically significant (e.g., *p* < .05).
